# Motion Planning of Autonomous Mobile Robot Using Recurrent Fuzzy Neural Network Trained by Extended Kalman Filter

**DOI:** 10.1155/2019/1934575

**Published:** 2019-01-29

**Authors:** Qidan Zhu, Yu Han, Peng Liu, Yao Xiao, Peng Lu, Chengtao Cai

**Affiliations:** College of Automation, Harbin Engineering University, Harbin 15001, China

## Abstract

This paper proposes a novel motion planning method for an autonomous ground mobile robot to address dynamic surroundings, nonlinear program, and robust optimization problems. A planner based on the recurrent fuzzy neural network (RFNN) is designed to program trajectory and motion of mobile robots to reach target. And, obstacle avoidance is achieved. In RFNN, inference capability of fuzzy logic and learning capability of neural network are combined to improve nonlinear programming performance. A recurrent frame with self-feedback loops in RFNN enhances stability and robustness of the structure. The extended Kalman filter (EKF) is designed to train weights of RFNN considering the kinematic constraint of autonomous mobile robots as well as target and obstacle constraints. EKF's characteristics of fast convergence and little limit in training data make it suitable to train the weights in real time. Convergence of the training process is also analyzed in this paper. Optimization technique and update strategy are designed to improve the robust optimization of a system in dynamic surroundings. Simulation experiment and hardware experiment are implemented to prove the effectiveness of the proposed method. Hardware experiment is carried out on a tracked mobile robot. An omnidirectional vision is used to locate the robot in the surroundings. Forecast improvement of the proposed method is then discussed at the end.

## 1. Introduction

In recent decades, unmanned ground mobile robots have been widely applied in various areas of both indoor and outdoor environments such as industry, mine, museum, port, or some dangerous places for their excellent maneuverability [1–3]. Research about navigation which can fully reflect artificial intelligence and automatic ability of unmanned ground mobile robots has been an attractive topic for a long time [4]. In order to achieve navigation, an effective motion planner should be designed [5]. Among the existing solutions, planning techniques were classified in two groups: nonheuristic methods and heuristic methods [6]. The most important nonheuristic methods consist of the potential field method (PFM) [7, 8], sampling-based planner (SBP), and interpolating curve method (ICM). PFM and SBP do not produce optimal paths and tend to be locked in some local minima [9]. ICM generates trajectories by constructing and inserting a new set of states considering reference points, i.e., a given set of way points, which cannot deal with dynamic surroundings well [10]. In order to solve the problem mentioned above, heuristic approaches are proposed. The most popular heuristic methods contain hybrid-heuristics A^*∗*^, neural network (NN), fuzzy logic (FL), genetic algorithm (GA), particle swarm optimization (PSO), etc. Contrasted to nonheuristic methods, heuristic methods are more intelligent and advanced to deal with complex problems [11]. However, the serious disadvantage is the necessary learning phase. Much online or offline computation is needed. So, a more efficient method should be proposed.

Fuzzy logic is well suited for programming mobile robot's motion for its accurate calculation capability and inference capability under uncertainty [12]. Many researchers have implemented this method to address the navigation problem of unmanned mobile robots. Wang and Liu [13] proposed a real-time fuzzy logic-based navigation strategy in unknown environments. The proposed approach employs a grid-based map that can record environment information and experience. However, the method focuses on building a map that is computationally expensive. The structure of fuzzy logic is so simple that it cannot deal with complex problems. Neural network is widely used due to its strong nonlinear approximation capability and self-learning capability [14]. Many researches have been done on the feedforward multilayer perception neural network [15]. However, feedforward NN methods require multilayer structures with a lot of neurons to represent dynamical responses. This leads to divergence and is time-consuming [16]. The weights of them are updated without considering the internal information and are sensitive to the training data. So, recurrent neural network attracts more attention for its superior dynamic capability. Recently, fuzzy logic and recurrent neural network structure are combined to form a new structure, i.e., recurrent fuzzy neural network (RFNN). Many approaches have been proposed by using RFNN and have shown superior performances. In [17], Juang et al. proposed a recurrent self-evolving interval type-2 fuzzy neural network for dynamic system processing. The structure forms a local internal feedback loop by feeding the firing strength of each rule back to itself. All rules are trained online via structure and parameter learning. Lin et al. [18] proposed an interactively recurrent fuzzy neural network for prediction and identification of dynamic systems. Their method is the same with Juang's method but employs a functional link neural network (FLNN) to the consequent part of fuzzy rules. The mapping ability is promoted. Although the concept of RFNN is investigated in detail, it has not been used in practical navigation well. For example, in [19], optimization of the result is not considered.

Back propagation (BP), evolutionary algorithm, and extended Kalman filter (EKF) are the three most popular training methods of supervised learning algorithms. In [20], the BP method is used to train the fuzzy neural network to achieve task planning and action selection of mobile robots. But, it needs data base and is trained offline. To apply RFNN to real-time nonlinear programs, an effective training method should be adopted. The extended Kalman filter is famous for its training efficiency and accuracy [21]. Rubio and Yu [22] applied EKF to train state-space recurrent neural networks, and identification of the nonlinear system is realized. And, the Lyapunov method is used to prove the stability of system. Wang and Huang [23] developed an effective RNN training approach based on EKF by using a controllable training convergence on the basis of Rubio. By adapting two artificial training noise parameters, i.e., the covariance of measurement noise and covariance of process noise, performance of EKF is improved. But, the proposed method is used in RNN instead of RFNN. The EKF algorithm possesses good online learning ability. Therefore, it is suitable for training RFNN to program the autonomous mobile robot's motion and achieving navigation.

Depending on the analysis above, the main contribution of this paper is that a real-time program strategy in unknown dynamic surroundings is proposed, i.e., without any previous offline computation. And, the optimal motion is generated in a free-space, i.e., without previous map information. A simple but effective RFNN structure is designed. A modified extended Kalman filter method is used to train RFNN in real time. An autonomous mobile robot is driven to reach the target and avoid obstacles. In the EKF training algorithm, target and obstacle constraints in practical situation are considered. Robustness of the proposed method against disturbances is discussed. Then, a numeric nonlinear optimization method and an update strategy are designed to guarantee robust optimization of the prediction. Besides the simulation experiment, our method is also evaluated on a real tracked mobile robot. An omnidirectional vision is used to locate the robot by using artificial landmarks on the basis of our previous work.

The rest of this paper is structured as follows.  Section 2 illustrates the modelling of the autonomous mobile robot surrounded by the target and obstacles.  Section 3 describes the planner in detail, including RFNN structure, EKF online learning algorithm, and convergence analysis.  Section 4 constructs the cost function and update strategy to guarantee optimization.  Section 5 is simulation and hardware experiment results. Finally, Section 6 concludes the proposed scheme.

## 2. Model of Autonomous Mobile Robot

Some programming methods ignore kinematic constraints [24]; thus, the stability of the system in practical situation cannot be guaranteed. An additional algorithm needs to be designed to smooth the trajectories. This leads to more computation cost. And, control effect of driving actual mobile robots to track the trajectory is not good. In order to get good dynamics performance in the tracking process, a natty kinematics of mobile robot is considered in motion planning.

**Figure 1:**
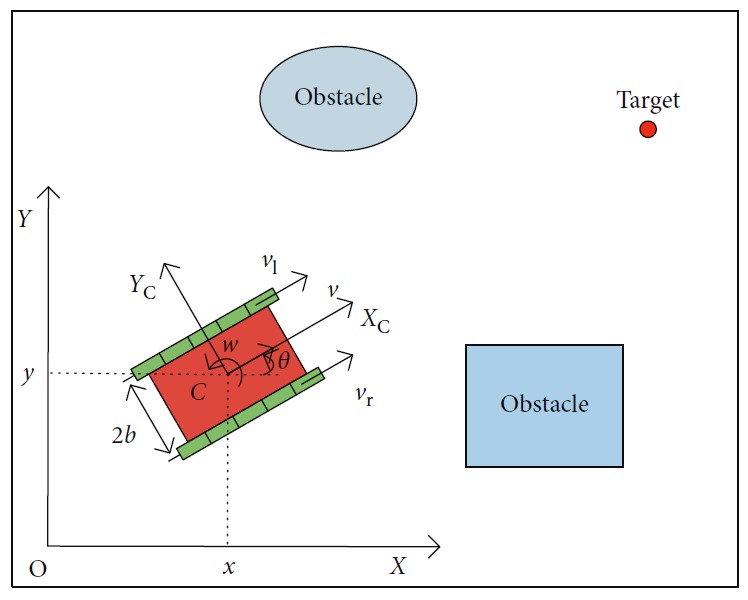
Model of the autonomous mobile robot in dynamic surroundings.

The autonomous mobile robot favored in this paper is a kind of tracked mobile platform. There are two caterpillar tracks independently driven by actuators for the mobile robot's motion, and they are placed symmetrically on both sides of the mobile robot. The kinematics can be illustrated as shown in Figure 1. *C* is the geometry center of a mobile platform. 2*b* is the length between two tracks. {*O*, *X*, *Y*} is the global coordinate frame, and {*C*, *X*_C_, *Y*_C_} is the local coordinate frame.

Assume that the unmanned mobile robot is made up of a rigid frame equipped with nondeformable caterpillar tracks. There is no slip between tracks and actuator gears. And, they are moving on a horizontal plane only in the direction normal to the axis of driving.

The posture is represented by three variables as

(1) $$\begin{array}{c} p={\left[x,y,\theta \right]}^{T}, \end{array}$$

where *x* and *y* are the coordinates of the center point *C* in {*O*, *X*, *Y*} and *θ* is the angle of its heading direction *X*_C_ taking counterclockwise from the *X*-axis. The motion state is

(2) $$\begin{array}{c} q={\left[v,w\right]}^{T}, \end{array}$$

where *v* is the linear velocity and *w* is the angular velocity of the mobile robot. Then, **p** and **q** can be associated by the following equitation:

(3) $$\begin{array}{c} p=\left[\begin{array}{cc} \text{cos }\theta & 0\\ \text{sin }\theta & 0\\ 0 & 1 \end{array}\right]q. \end{array}$$

Thr autonomous mobile robot is motivated by a pair of independent caterpillar tracks, so

(4) $$\begin{array}{c} v=\frac{\left(v_{\text{r}}+v_{\text{l}}\right)}{2}, \end{array}$$

(5) $$\begin{array}{c} w=\frac{\left(v_{\text{r}}-v_{\text{l}}\right)}{2b}, \end{array}$$

(6) $$\begin{array}{c} R=\frac{b\left(v_{\text{r}}+v_{\text{l}}\right)}{\left(v_{\text{r}}-v_{\text{l}}\right)}, \end{array}$$

where *v*_r_ is the right linear velocity, *v*_l_ is the left linear velocity, and *R* is the rotation radius of the mobile robot.

A simple discrete-time kinematic model is used in this paper to illustrate the moving process. The difference equation can be illustrated as

(7) $$\begin{array}{c} p_{k+1}=p_{k}+\Delta t\left[\begin{array}{cc} \text{cos }\theta_{k} & 0\\ \text{sin }\theta_{k} & 0\\ 0 & 1 \end{array}\right]q_{k}. \end{array}$$

The positions of the target and obstacles in the global coordinate frame need to be transformed to the local frame. The transformation matrix is

(8) $$\begin{array}{c} T=\left[\begin{array}{ccc} \text{cos }\theta & \text{sin }\theta & -x\text{ cos }\theta -y\text{ sin }\theta \\ -\text{sin }\theta & \text{cos }\theta & x\text{ sin }\theta -y\text{ cos }\theta \\ 0 & 0 & 1 \end{array}\right]. \end{array}$$

## 3. Motion Planner Based on RFNN

**Figure 2:**
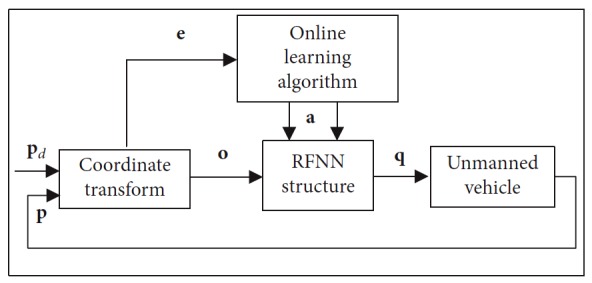
Nonlinear program strategy based on RFNN.

A nonlinear program strategy is shown in Figure 2. It is made up of four parts such as coordinate transformation, RFNN structure, unmanned mobile robot model, and online learning algorithm. The coordinate transformation part establishes the environment map. Target and obstacles' information is then collected. The RFNN structure generates desired velocities of the mobile robot. It is trained by the extended Kalman filter that makes up the online learning algorithm. Detailed information of each part is shown in Figure 2.

### 3.1. RFNN Structure

**Figure 3:**
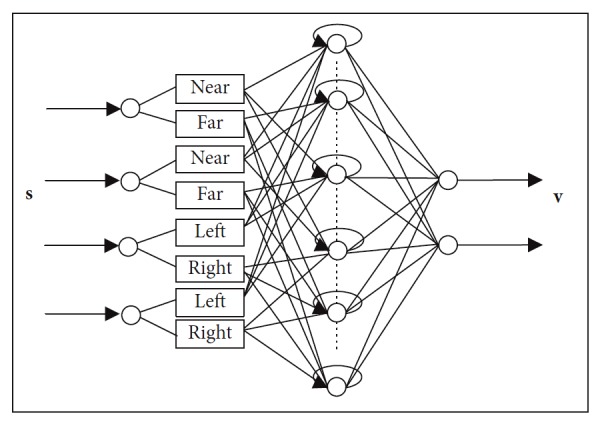
Structure of RFNN.

A simple recurrent fuzzy neural network is designed in this section for the purpose of improving computation efficiency. Considering that the navigation system is multiinput multioutput, RFNN is made up of five layers as shown in Figure 3. The structure is first used in our previous work [25]. In this paper, detailed information of the structure and training progress is introduced. Convergence is analyzed. Furthermore, the original structure is improved in this paper to achieve nonlinear motion planning.

#### 3.1.1. Layer 1 (Input Layer)

Only the current state is traded as the input in this layer. **s**=(*s*_1_, *s*_2_, *s*_3_, *s*_4_)=(*d*_g_, *d*_o_, *θ*_g_, *θ*_o_) is chosen as the input, so there are 4 nodes transmitting the input variables to the next layer directly. *d*_g_ is the distance between mobile robot and goal. *d*_o_ is the distance between mobile robot and the nearest obstacle. *θ*_g_ is the angle between mobile robot's front direction and goal. *θ*_o_ is the angle between mobile robot's front direction and the nearest obstacle.

#### 3.1.2. Layer 2 (Membership Layer)

Each node in this layer performs a membership function. In this paper, the input of the membership layer is *s*_*i*_ (*i*=1,2,3,4). The membership function is defined with Gaussian MF as follows:

(9) $$\begin{array}{c} {\text{net}}_{j}\left(s_{i}^{j}\right)=-\frac{{\left(s_{i}^{j}-c_{i}^{j}\right)}^{2}}{{\left(\sigma_{i}^{j}\right)}^{2}}, \end{array}$$

(10) $$\begin{array}{c} u_{i}^{j}\left[{\text{net}}_{j}\left(s_{i}^{j}\right)\right]=\exp\left[{\text{net}}_{j}\left(s_{i}^{j}\right)\right], \end{array}$$

where exp[·] is the exponential function and *c*_*i*_^*j*^ and *σ*_*i*_^*j*^ (*j* is the number of the linguistic variables with respect to each input) are the mean and standard deviation of the Gaussian function, respectively. The values of them are initially set via expert experience before the strategy begins.

According to the actual occasion, *d*_g_ (*d*_o_) can be divided into far and near depending on the distance between mobile robot and goal (the nearest obstacle). *θ*_g_ (*θ*_o_) can be divided into left and right depending on goal's position (the nearest obstacle's position) related to the mobile robot's front direction. So, there are two linguistic variables of each input.

#### 3.1.3. Layer 3 (Fuzzy Rule Layer)

Each node in this layer corresponds to one fuzzy rule. As shown in Figure 3, each input has two membership values. Hence, there are 16 fuzzy rules.

The firing strength of each rule at the current step is determined by the outputs of layer 2 through an AND operator. The result of each rule is calculated as follows:

(11) $$\begin{array}{c} f_{n}=\prod u_{i}^{j},\;\left(i=1,2,3,4,\;\;j=1,2,\;\;n=1,2,\ldots ,16\right). \end{array}$$

Moreover, a local internal feedback with a time delay is added to each node of this layer forming a recurrent frame. The mathematical form is described as

(12) $$\begin{array}{c} \psi_{nk}=\left(1-\lambda_{n}\right)f_{n}+\lambda_{n}\psi_{n\left(k-1\right)}, \end{array}$$

where *λ*_*n*_ is the constant representing weight of a self-feedback loop and *ψ*_*n*(*k* − 1)_ indicates the output of layer 3 in the previous time step.

#### 3.1.4. Layer 4 (Consequent Layer)

This layer describes a linear combination of functions in the consequent part, and each node is called the consequent node. According to the definition of TSK fuzzy rules, weight *w*_*o*_=(*w*_*o*1_, *w*_*o*2_,…, *w*_*on*_) can be obtained:

(13) $$\begin{array}{c} w_{on}=a_{on}^{1}s_{1}+a_{on}^{2}s_{2}+\cdots +a_{on}^{i}s_{i}. \end{array}$$

For the purpose of simplicity calculation, it is assumed that *a*_*on*_=*a*_*on*_^1^=⋯=*a*_*on*_^*i*^. So, *w*_*on*_=*a*_*on*_(*s*_1_+*s*_2_+*s*_3_+*s*_4_), and *o*=1,2.

The output of this layer is

(14) $$\begin{array}{c} \varphi_{o}=\underset{n}{\sum }w_{on}\psi_{n}. \end{array}$$

#### 3.1.5. Layer 5 (Output Layer)

There are two nodes in this layer representing linear velocities of right and left caterpillar tracks, respectively. An activation function is set at each node:

(15) $$\begin{array}{c} v_{o}=\frac{\varphi_{o}}{\left(1+\alpha \left|\varphi_{o}\right|\right)}, \end{array}$$

where *α* is a constant.

### 3.2. Online Training Algorithm Based on EKF

The EKF training algorithm can be summarized as parallel EKF and parameter-based EKF [26]. In this paper, the parameter-based EKF in [23] is modified to learn weights of RFNN. Considering the practical condition in motion planning of an autonomous mobile robot, a Jacobian matrix is designed.

At time step *k*, the EKF function has the following form:

(16) $$\begin{array}{c} a_{k+1}=a_{k}-K_{k}e_{k},\\ e_{k}=o_{k}-o_{dk},\\ K_{k}=P_{k}O_{k}{\left(R_{k}+O_{k}^{T}P_{k}O_{k}\right)}^{-1},\\ P_{k+1}=Q_{k}+\left(I-K_{k}O_{k}^{T}\right)P_{k}, \end{array}$$

where **a**_*k*_=(*a*_11_,…,*a*_1,16_, *a*_21_,…,*a*_2,16_)^*T*^ is the estimation of weights, **K** is the Kalman gain matrix, **e** is the estimation error, **o**_*d*_ is the desired value of **o** which is the observation vector, **O** is the orderly derivative matrix, **R** is the covariance matrix of the measurement error, **Q** is the covariance matrix of the process noise, **P** is the covariance matrix of the estimation error, and **I** is the identity matrix.

In order to achieve navigation, both distance and angle information need to be considered. So, the observation vector can be represented as **o**=(*d*_g_, *d*_o_, *θ*_g_, *θ*_o_)^*T*^.

Then, **O** can be calculated as

(17) $$\begin{array}{c} O=\frac{\partial o^{T}}{\partial a}. \end{array}$$

As the only one-step recurrence is considered here, we take *d*_g_ as an example to calculate **O**. Then, it is the same to *d*_o_, *θ*_g_,  and *θ*_o_:

(18) $$\begin{array}{c} d_{\text{g}k}=\sqrt{{\left(x_{k}-x_{d}\right)}^{2}+{\left(y_{k}-y_{d}\right)}^{2}}=\sqrt{x_{dk}^{\prime 2}+y_{dk}^{\prime 2}}, \end{array}$$

where (*x*_*d*_′, *y*_*d*_′, 1)^*T*^=*T*(*x*_*d*_, *y*_*d*_, 1)^*T*^ is the target position in the local frame.

Then,

(19) $$\begin{array}{c} \frac{\partial d_{gk}}{\partial a}=\frac{\left[\left(x_{k}-x_{d}\right)\partial x_{k}/\partial a+\left(y_{k}-y_{d}\right)\partial y_{k}/\partial a\right]}{d_{gk}}, \end{array}$$

and according to (7),

(20) $$\begin{array}{c} \frac{\partial x_{k}}{\partial a}=\frac{\partial x_{k-1}}{\partial a}+\text{cos }\theta_{k-1}\frac{\partial v_{k-1}}{\partial a}-v_{k-1}\text{sin }\theta_{k-1}\frac{\partial \theta_{k-1}}{\partial a},\\ \frac{\partial y_{k}}{\partial a}=\frac{\partial y_{k-1}}{\partial a}+\text{sin }\theta_{k-1}\frac{\partial w_{k-1}}{\partial a}+w_{k-1}\text{cos }\theta_{k-1}\frac{\partial \theta_{k-1}}{\partial a},\\ \frac{\partial \theta_{k}}{\partial a}=\frac{\partial \theta_{k-1}}{\partial a}+\frac{\partial w_{k-1}}{\partial a}, \end{array}$$

and according to (4), (5), and (24),

(21) $$\begin{array}{c} \frac{\partial v_{k-1}}{\partial a}=\frac{\psi_{n\left(k-1\right)}\sum s_{k-1}}{2{\left(1+\alpha \varphi_{o\left(k-1\right)}\right)}^{2}},\\ \frac{\partial w_{k-1}}{\partial a}=\pm \frac{\psi_{n\left(k-1\right)}\sum s_{k-1}}{2b{\left(1+\alpha \varphi_{o\left(k-1\right)}\right)}^{2}}, \end{array}$$

where *o*=1,2 and *n*=1,2,…, 16 corresponding to **a**=(*a*_11_,…,*a*_1,16_, *a*_21_,…,*a*_2,16_)^*T*^. If *o*=1, the results are positive. Otherwise, the results are negative.

**Figure 4:**
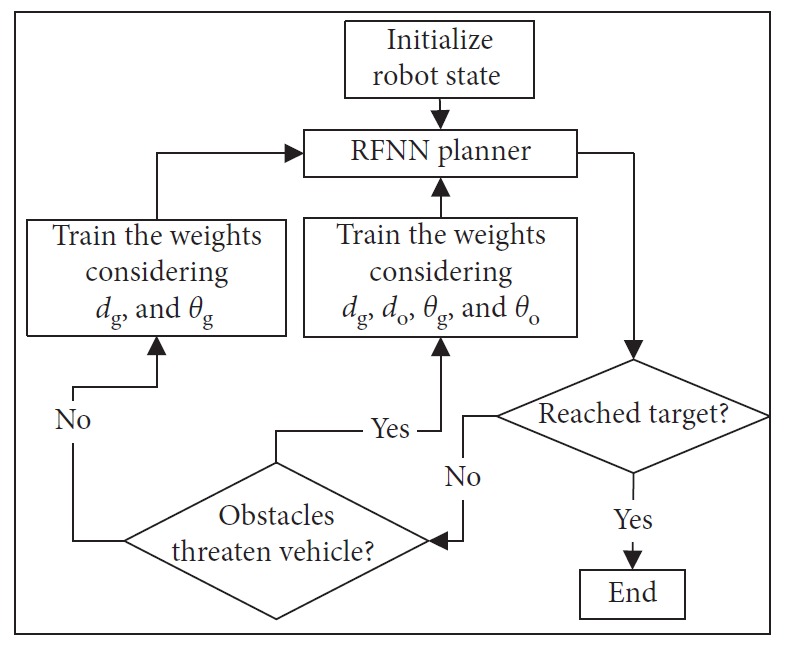
Flow diagram of EKF.

If the distance between obstacles is long enough for the mobile robot to pass through without collision, *d*_o_ and *θ*_o_ will not be considered in EKF. However, if the distance is shorter than safe distance, the EKF algorithm should train the weights of RFNN to avoid collision. *d*_o_ and *θ*_o_ are then considered at this moment. The flow diagram is shown in Figure 4.

### 3.3. Convergence Analysis

In this section, we will prove that the EKF proposed in the above section is effective to RFNN in Section 3.1. And the designed Jacobian matrix **O** is reasonable and feasible.

As shown in Figure 2, observation vector **o** is a function of **v**, i.e., **o**=*𝒪*(**v**). By calculating the first-order derivative of **a** in (21), fuzzy logic inference and weights learning process are clearly reflected in **O**. For the parameter-based EKF, only weights are viewed as states to be estimated [23]. **o** is first expanded at optimal weights **a**_*d*_ as

(22) $$\begin{array}{c} o_{k}=O\left(a_{d}\right)+\left(a_{k}-a_{d}\right)\frac{\partial o}{\partial a}+\xi_{k}, \end{array}$$

where **ξ**_*k*_ is the first-order approximation residue. The error of weights can be defined as **e**_*ak*_=**a**_*k*_ − **a**_*d*_.

Then, the Lyapunov function is written as

(23) $$\begin{array}{c} E_{k}=e_{ak}^{T}P_{k}^{T}e_{ak},\\ \Delta E_{k}=E_{k+1}-E_{k}. \end{array}$$

Then,

(24) $$\begin{array}{c} \Delta E_{k}=e_{a\left(k+1\right)}^{T}P_{k+1}^{-1}e_{a\left(k+1\right)}-e_{ak}^{T}P_{k}^{-1}e_{ak}<{\left[e_{a\left(k+1\right)}-e_{ak}\right]}^{T}P_{k}^{-1}e_{ak}-e_{a\left(k+1\right)}^{T}{\left[P_{k+1}-Q_{k}\right]}^{-1}P_{k}O_{k}B_{k}^{-1}\xi_{k}. \end{array}$$

According to Jolly et al. [20], (24) becomes

(25) $$\begin{array}{c} \Delta E_{k}<\frac{-{\left\|e_{k}\right\|}^{2}}{o_{k}/m+r_{k}}+\frac{3{\left\|\xi_{k}\right\|}^{2}}{r_{k}}, \end{array}$$

where **o**_*k*_ is the trace of **O**_*k*_^*T*^**P**_*k*_**O**_*k*_. *m* is the dimension of **I** in (16), and *r*_*k*_ is a positive real number. As mentioned in the above section, the dimension of **O** changes depending on the position of obstacles related to the mobile robot. According to the calculate trace **o**_*k*_, the jumping change will not affect the stability of the training process.

From (25), we can see that the convergence of the training process is determined by **e**_*k*_, **o**_*k*_, and *r*_*k*_. In order to guarantee Δ*E*_*k*_ < 0, *r*_*k*_ should be set as

(26) $$\begin{array}{c} r_{k}>\frac{3o_{k}{\left\|\xi_{k}\right\|}^{2}}{m\left({\left\|e_{k}\right\|}^{2}-3{\left\|\xi_{k}\right\|}^{2}\right)}. \end{array}$$

If ${\left\|e_{k}\right\|}^{2}<4{\bar{\xi }}_{k}^{2}$ (${\bar{\xi }}_{k}\geq \left\|\xi_{k}\right\|$), the error is bounded and the process is convergent.

If ${\left\|e_{k}\right\|}^{2}>4{\bar{\xi }}_{k}^{2}$, the inequality will become

(27) $$\begin{array}{c} r_{k}>\frac{3o_{k}}{m}. \end{array}$$

If each element of **ξ**_*k*_ is of normal distribution, *ξ*_*ik*_ ~ *N*(0, *r*_*k*_), then,

(28) $$\begin{array}{c} {\left\|e_{k}\right\|}^{2}>4{\bar{\xi }}_{k}^{2}=64r_{k}m, \end{array}$$

where 99.99%*ξ*_*ik*_ are bounded. Then,

(29) $$\begin{array}{c} \frac{3o_{k}}{m}<r_{k}<\frac{{\left\|e_{k}\right\|}^{2}}{64m}. \end{array}$$

*r*_*k*_ can be chosen as

(30) $$\begin{array}{c} r_{k}=\frac{\left({\left\|e_{k}\right\|}^{2}/64m+3o_{k}/m\right)}{2}. \end{array}$$

Convergence of the training process is guaranteed, i.e., bounded **e**_*k*_.

## 4. Trajectory Optimization

As mentioned above, feasible trajectories are generated. But, these trajectories are always suboptimal and worthy of further improvement. So, in this section, the numerical optimization procedure is designed to obtain the optimal trajectory. Considering the practical situation, power consumption, driving distance, and time are favored to determine optimization of the trajectory. A new variable object is established as Tra={**p**, **v**, **w**}, indicating that each trajectory is represented by the mobile robot's state and linear and angular velocities.

Then, the objective function is structured as

(31) $$\begin{array}{c} J=W_{1}\overset{N^{l}}{\underset{k=1}{\sum }}{\left\|p_{k}^{l}-p_{k-1}^{l}\right\|}^{2}+W_{2}\overset{N^{l}}{\underset{k=1}{\sum }}{\left\|v_{k}^{l}-{\bar{v}}^{l}\right\|}^{2}+W_{3}\overset{N^{l}}{\underset{k=1}{\sum }}{\left\|w_{k}^{l}-{\bar{w}}^{l}\right\|}^{2}+W_{4}N^{l}, \end{array}$$

where **W** is the weight of each item. The first and last items in (31) represent moving distance and time, respectively. Furthermore, we want the motion of the mobile robot to be smooth in the dynamic environment. So, the second and third items are designed in (31). $\bar{v}$ and $\bar{w}$ are the mean values.

The autonomous mobile robot is driven by the target and needs to arrive at destination in limited area. During the process, obstacle avoidance is considered. Then, the target and obstacle constraints are

(32) $$\begin{array}{c} {\left\|p_{\text{f}}-p_{\text{t}}\right\|}_{2}\leq \Delta_{t}, \end{array}$$

(33) $$\begin{array}{c} {\left\|p_{k}-p_{\text{o}}\right\|}_{2}\geq \Delta_{o}, \end{array}$$

where **p**_f_ is the final state of the mobile robot, **p**_*k*_ is the state at each step, **p**_t_ is the target state, and **p**_o_ is the obstacle state. These are achieved according to (16), (17), and (21).

In the practical application, the linear and angular velocities of the mobile robot are limited, so

(34) $$\begin{array}{c} 0\leq q_{k}\leq q_{\text{max}}, \end{array}$$

which is achieved according to (9)–(15).

Then, the optimization problem becomes

(35) $$\begin{array}{c} {\text{Tra}}^{*}=\underset{\text{Tra}}{\arg\text{ min}}J. \end{array}$$

If the dynamic environment can be predicted or motion of the obstacle is not too drastic, our method is able to predict optimal trajectory without supplement. This is analyzed in the simulation experiment below. However, the dynamic environment is unpredicted and motion of the obstacle is irregular. So, an extra algorithm should be designed.

In order to update the trajectory online, the detection of data in real time should be carried out. The following variable is established to measure changes in the environment:

(36) $$\begin{array}{c} \sigma ={\left(o_{k}^{d}-o_{k}^{m}\right)}^{T}W_{5}\left(o_{k}^{d}-o_{k}^{m}\right), \end{array}$$

where **o**_*k*_^*d*^ is the detection of the observation vector at each step and **o**_*k*_^*m*^ is the memory value of the current trajectory. **W**_5_ is the matrix of weights. The upper bound is set

(37) $$\begin{array}{c} \sigma <\sigma_{\text{bound}}. \end{array}$$

## 5. Experiments

### 5.1. Motion Planning Based on RFNN

To prove the effectiveness of the proposed motion planning method, the simulation experiment is carried out in this section using the Matlab software. And, all experiments are performed on a computer with Intel i5 2.3 GHz processor and 8 GB RAM. The simulation area is limited in 10  m × 10  m. There contain obstacles and target. The autonomous mobile robot needs to reach the target and avoid obstacles. The detailed values of the variables that need to be set manually are listed in Table 1.

**Figure 5:**
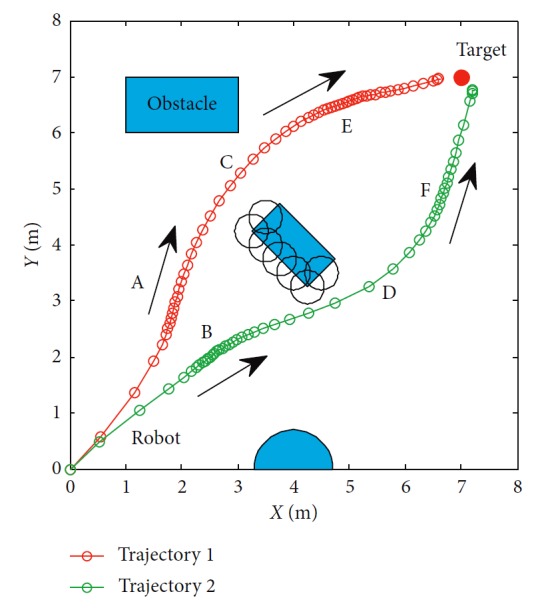
Motion trajectory of the mobile robot.

As illustrated in Figure 4, the navigation method proposed here is goal driven. The program strategy generates the trajectory guiding autonomous mobile robot to move from the starting point to the target. At each step, only the nearest position of the obstacle that threatens the mobile robot's safety is used in the training process of RFNN. So, the obstacle model is established using points at the edge as shown in Figure 5. Circle represents the dangerous region whose radius is related to the error term in (16). It is determined by the EKF algorithm's training speed. Distance between centers of adjacent circles depends on *b*. If it is too long, the model becomes invalid. If it is too short, the model is too compact to waste much time in computation. When a suitable distance is chosen, a sparse representation of the obstacle is established. Motion planning is first carried out in the static environment. The target and obstacles are supposed to be fully detected in real time.

**Figure 6:**
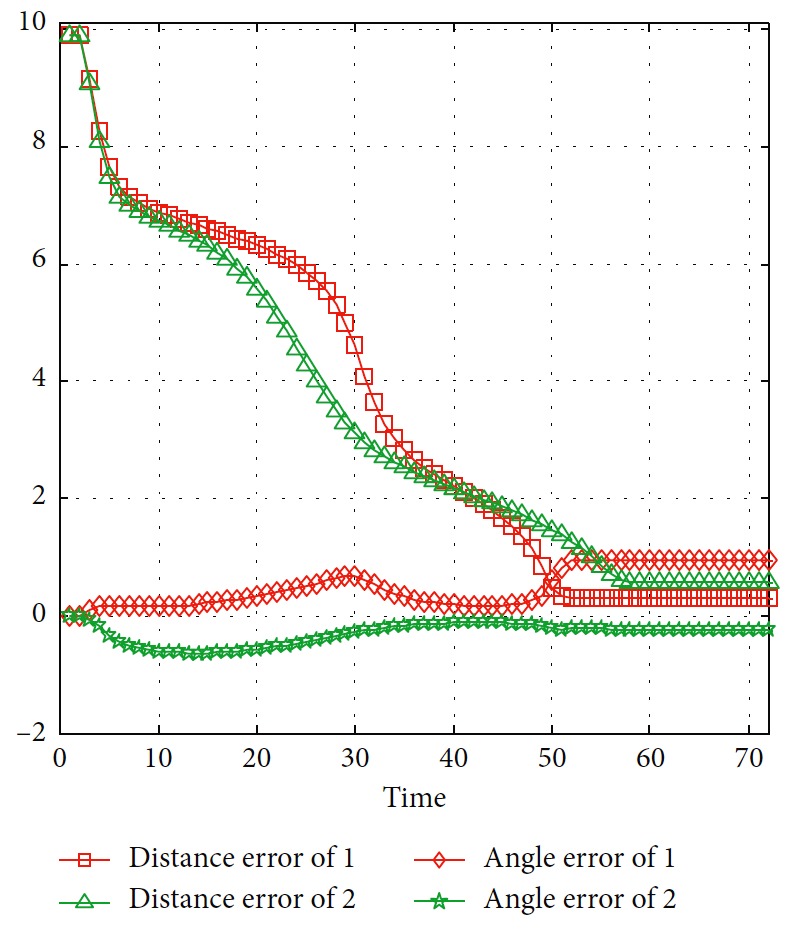
Distance and angle error of the mobile robot.

**Figure 7:**
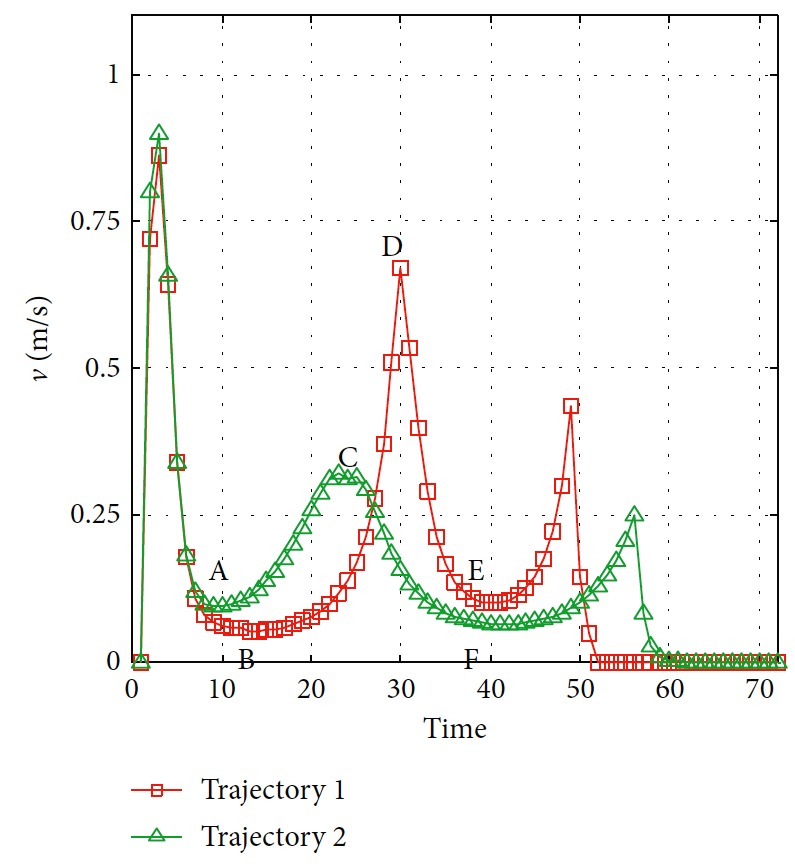
Linear velocity of the mobile robot.

**Figure 8:**
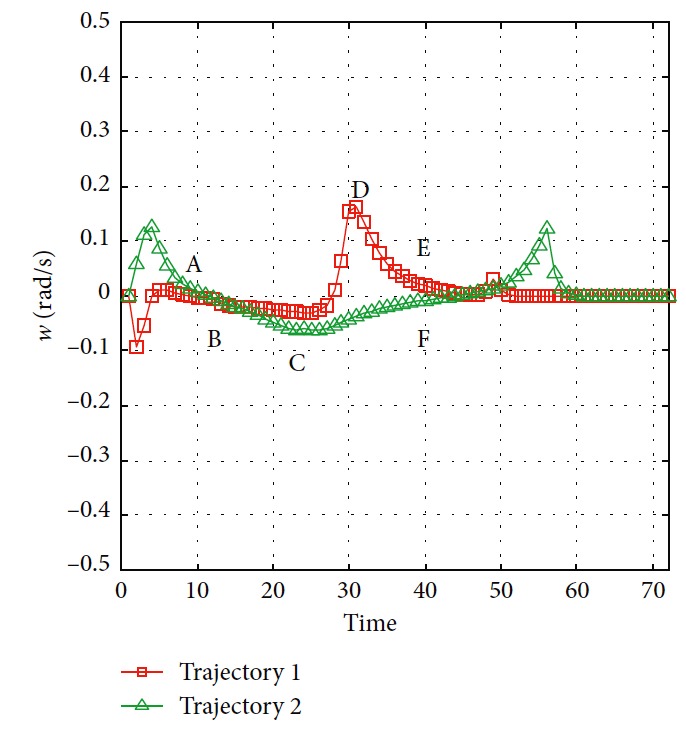
Angular velocity of the mobile robot.

**Figure 9:**
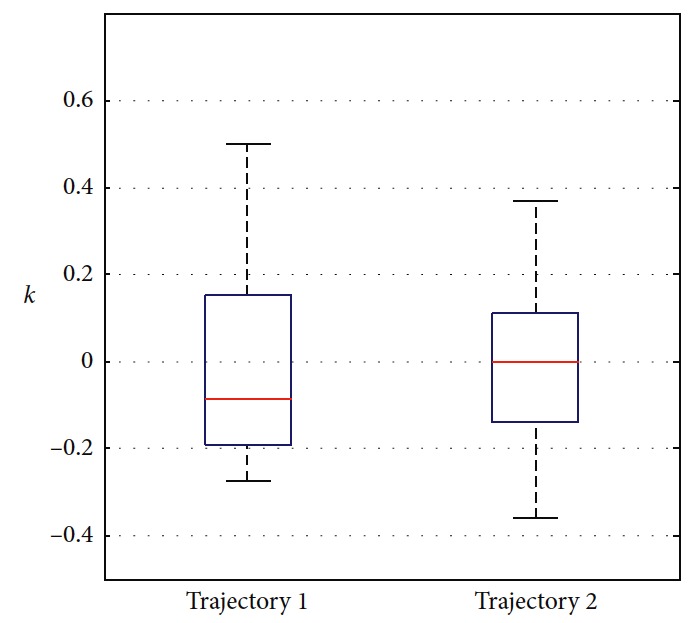
Statistical analysis of the curvature.

The trajectories without the numerical optimization are shown in Figure 5. The two motion trajectories are listed here. Each mark on the path represents the planned position of the mobile robot at each step. Changes of motion are reflected clearly. The position error of the robot during the process is shown in Figure 6. When the robot moves close to the obstacle, it slows down at points A and B. This satisfies the actual requirement and guarantees safety. The corresponding linear velocity is shown at points A and B in Figure 7. The corresponding angular velocity is shown at points A and B in Figure 8. When the robot avoids the collision with the obstacle, it speeds up to shorten time to arrive the target. The corresponding linear velocity is shown at points C and D in Figure 7. The corresponding angular velocity is shown at points C and D in Figure 8. At points E and F, the robot slows down to arrive the target. The statistical analysis of the curvature is shown in Figure 9. It can be seen that the programmed motion is smooth and is fit to the actual action.

Information of the two trajectories is shown in Table 2. Distance, step, cost, and terminal error are chosen as evaluation criteria in this paper. In order to observe the state of RFNN during process, weights are listed in Tables 3 and 4. The weights are initially set in random. They keep changing during the process to drive the robot to the target and avoid obstacles. From *k*=40 to *k*=50, we can see that they are convergent at the destination.

### 5.2. Optimization of Trajectory

**Figure 10:**
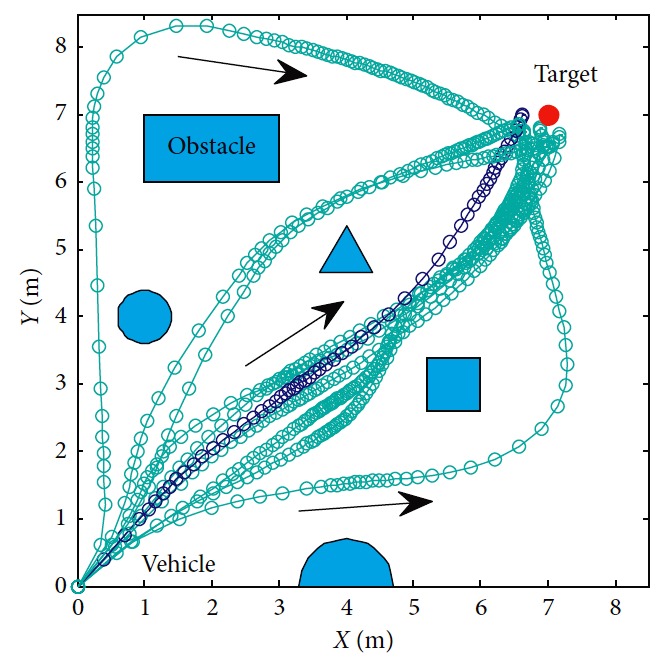
Motion trajectories of the mobile robot.

**Figure 11:**
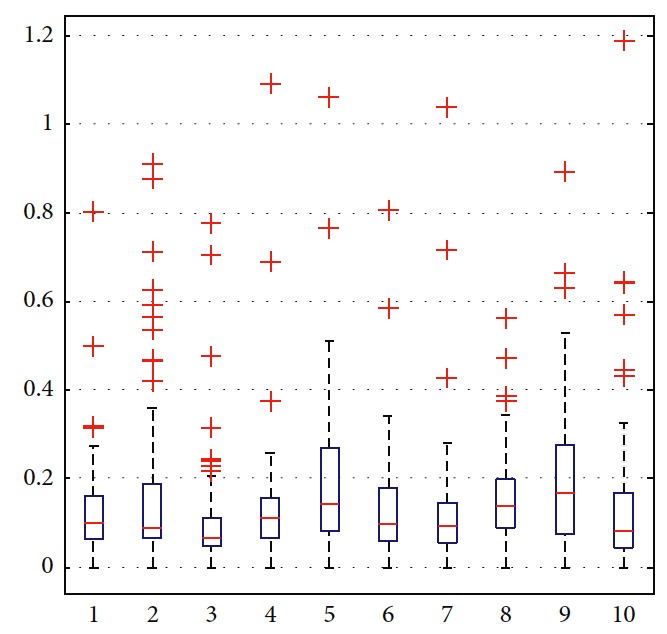
Statistical analysis of velocity.

**Figure 12:**
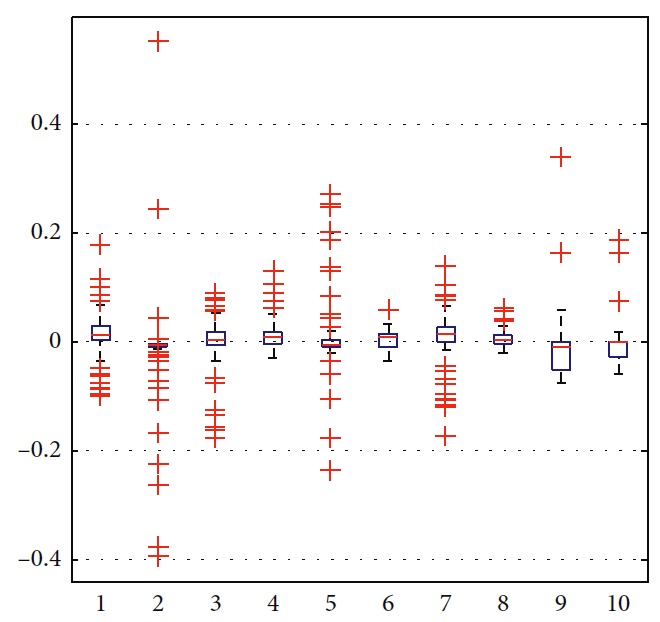
Statistical analysis of angular velocity.

**Figure 13:**
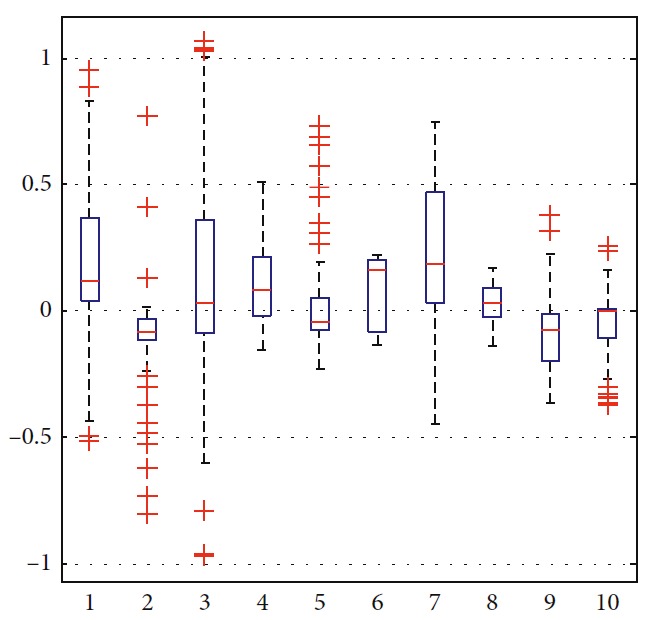
Statistical analysis of the curvature.

In order to illustrate the optimization of the solution, the trajectories are generated as shown in Figure 10. Among all trajectories, only the blue one is generated considering (31)–(35). The corresponding number in Table 5 is eight. The left nine cyan trajectories are generated randomly. Detailed information is listed in Table 5. By suitably choosing weights in (31), moving distance, step, and smoothness of trajectory are taken into comprehensive consideration. It can be seen that more computation is needed to generate optimal trajectory. The terminal error can be reduced by setting the target constraint in (32). In this paper, it is set as 0.5. As shown in the boxplot of velocity in Figures 11 and 12, the values of the eighth one are more concentrated and outliers are smaller. The performance of the eighth motion trajectory is better than that of others, which is reflected by the curvature in Figure 13.

### 5.3. Comparison with Other Methods

As mentioned above, there are many programming methods. In order to prove our method's effectiveness, other methods are compared here. OPTI supports for solving optimal problems and consists of popular optimization solvers. So, a numerical planner using the nonlinear program solvers in OPTI is designed. Kinematic constraint, target, and obstacle constraints are considered in the process. Furthermore, the classical A^*∗*^ method which is carried out using the grid map is also taken into comparison.

**Figure 14:**
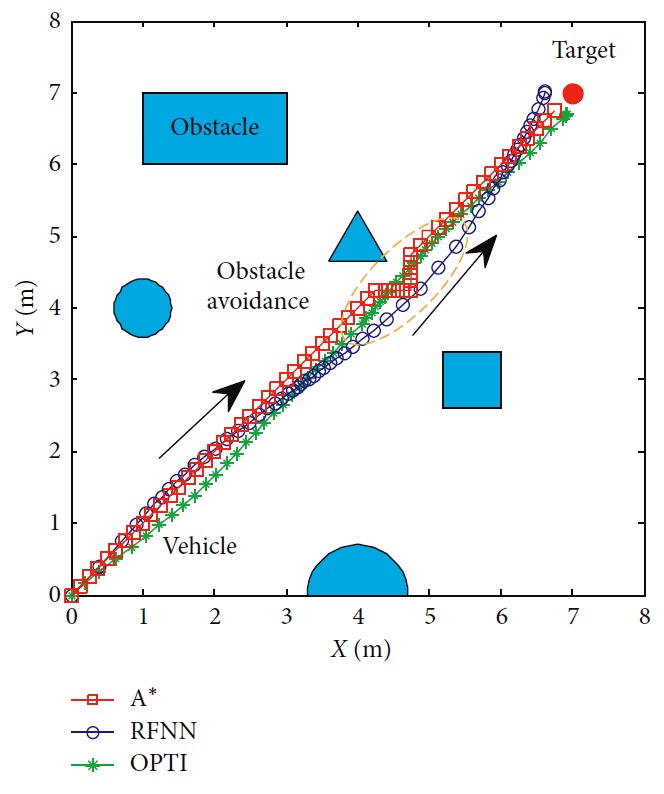
Motion trajectories of the mobile robot.

**Figure 15:**
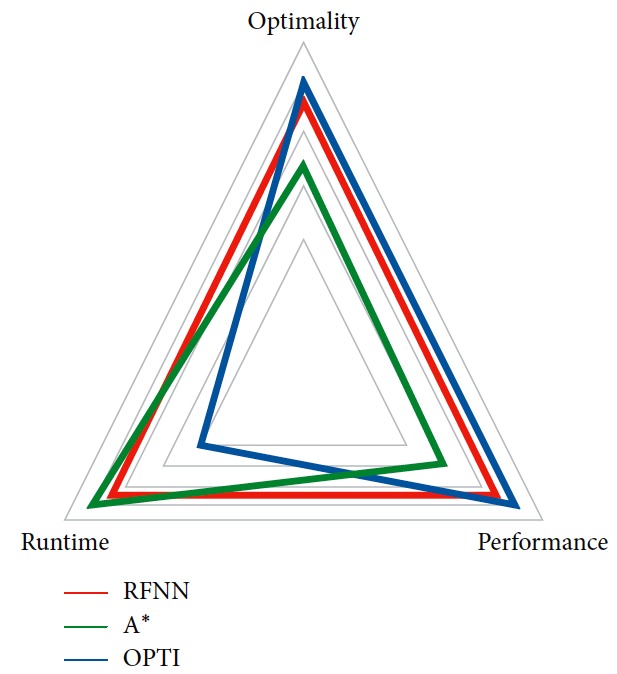
Qualitative comparison.

The results are shown in Figure 14. The numerical method is continuous in motion for the reasons that it plans the control command instead of the mobile robot's position. And, the state of the mobile robot is space free. A^*∗*^ plans only the position of the mobile robot and the path is not smooth. An extra controller considering kinematic constraints of the mobile robot should be added to track the path. Because it is based on the grid map, the planned path can be unsuitable for the robot to follow. A big curvature makes performance bad, such as the orange circle area in Figure 14. Although the performance of the numerical method is better, it takes up more computation time than A^*∗*^ as listed in Table 6. Compared to these two kinds of methods, our planner is continuous in motion and the generated trajectory is smooth. Computation time is much shorter than the numerical method to achieve the same performance, but it is a little longer than A^*∗*^. The qualitative comparison is illustrated in Figure 15.

### 5.4. Robustness of RFNN Planner

In this paper, we want to realize real-time motion planning. So, robustness has to be guaranteed. The weights of RFNN are trained online. Performance depends on initial weights. After initial weights are ensured, trajectory is fixed under the current condition. However, in the practical situation, dynamic environment and perception inaccuracy influence performance of motion planning. So, in this section, influence of these factors to our planning method is introduced.

**Figure 16:**
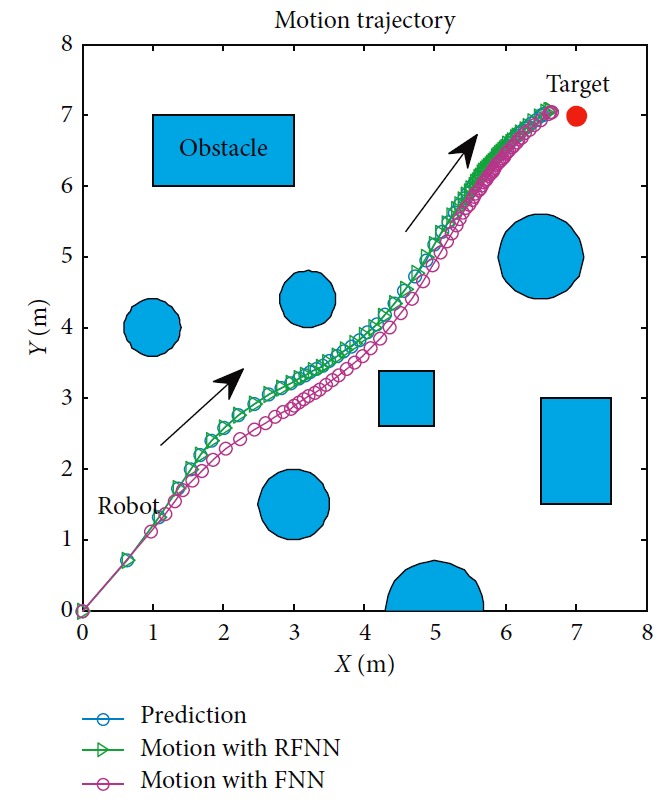
Qualitative comparison.

In order to prove the robustness of RFNN, the FNN-based planner is compared in this section first. Initial weights **a**_0_ of RFNN and FNN are both set as [0.1057, 0.1420, 0.1664, 0.6209, 0.5737, 0.0520, 0.9312, 0.7286, 0.7378, 0.0634, 0.8604, 0.9344, 0.9843, 0.8589, 0.7855, 0.5133, 0.1776, 0.3985, 0.1339, 0.0308, 0.9391, 0.3013, 0.2955, 0.3329, 0.4670, 0.6481, 0.0252, 0.8422, 0.5590, 0.8540, 0.3478, 0.4460]. It is supposed that there is a perception error. In the actual motion period, the obstacles' position is biased contrasted to the prediction period. The performance is shown in Figure 16. Motion with RFNN under the perception error is the same as that under prediction. The recurrent frame in RFNN structure improves the robustness of the planner. On the contrary, motion with FNN is the biased contrasted prediction. This can cause dangerous in the actual situation.

**Figure 17:**
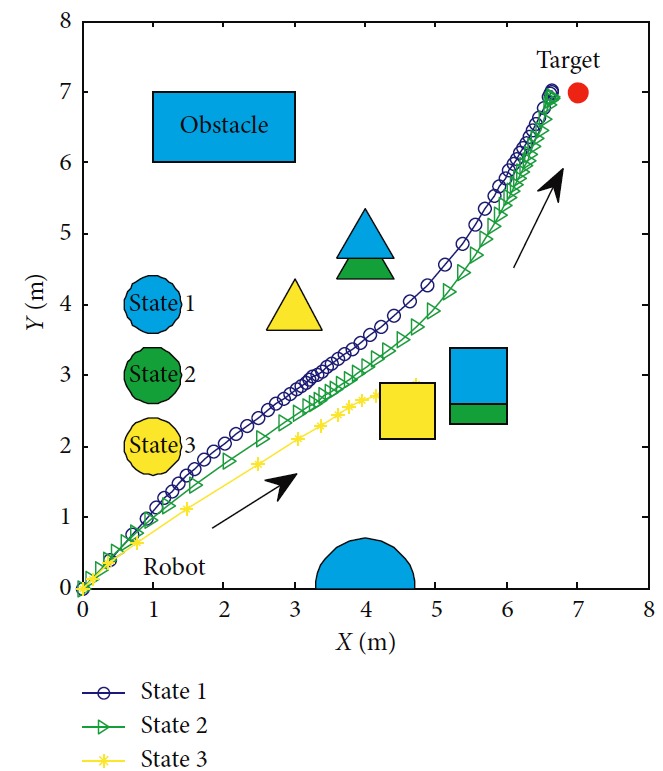
Motion trajectory of the mobile robot.

**Figure 18:**
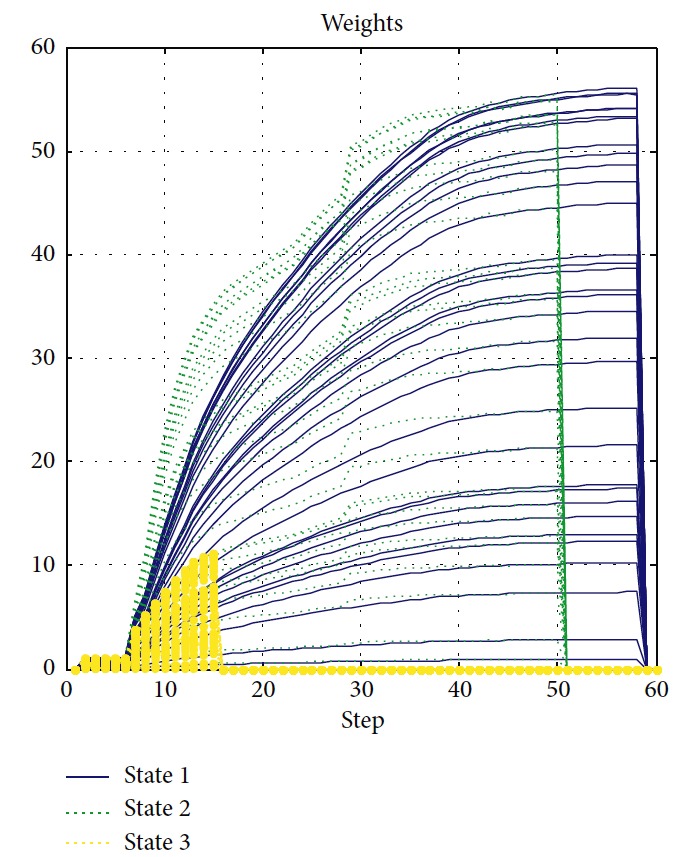
Motion trajectory of the mobile robot.

To introduce our method's robustness in dynamic surroundings, the experiment is carried out as shown in Figure 17. Initial weight **a**_0_ is set as [0.4401, 0.6305, 0.2144, 0.6398, 0.9684, 0.6972, 0.8841, 0.9268, 0.9452, 0.5178, 0.1781, 0.8219, 0.0489, 0.1291, 0.9319, 0.2809, 0.9441, 0.6843, 0.6741, 0.3766, 0.8681, 0.5585, 0.3033, 0.8492, 0.7845, 0.9706, 0.9782, 0.6032, 0.0149, 0.2569, 0.3101, 0.2272]. We assume that surroundings change to states 2 and 3. Corresponding trajectories are green and yellow, respectively. The changes of RFNN weights of each condition are, respectively, illustrated in Figure 18. Detailed information of each trajectory is shown in Table 7. It proves that our method possesses robustness in the dynamic environment with little amplitude changes. It loses efficacy if the changes are drastic. So, the dynamic update mechanism of initial weights of RFNN should be designed necessarily.

### 5.5. Update of Motion Trajectory

**Figure 19:**
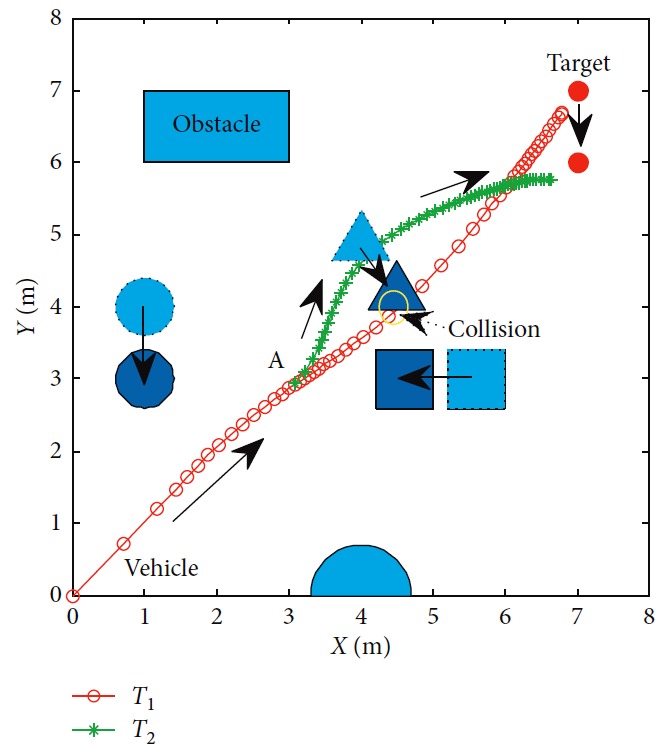
Motion trajectory of the mobile robot.

**Figure 20:**
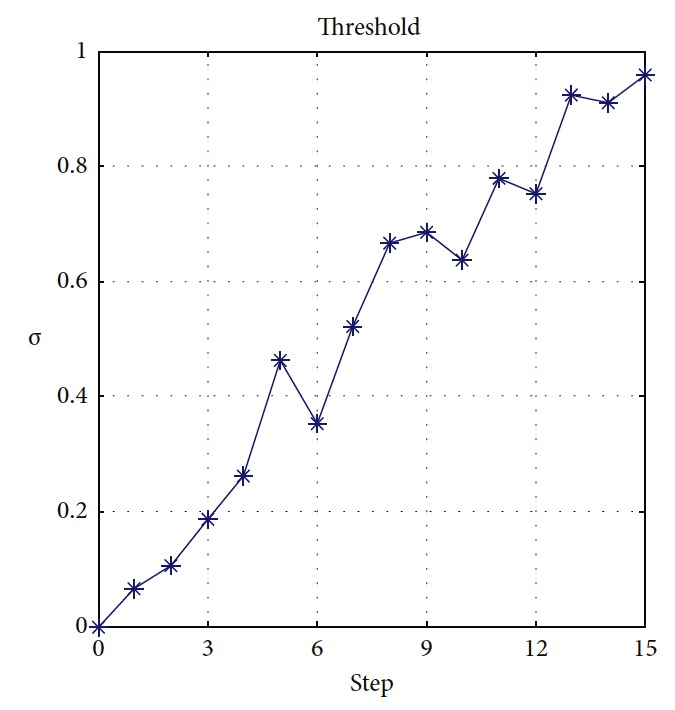
Changes of *σ*.

**Figure 21:**
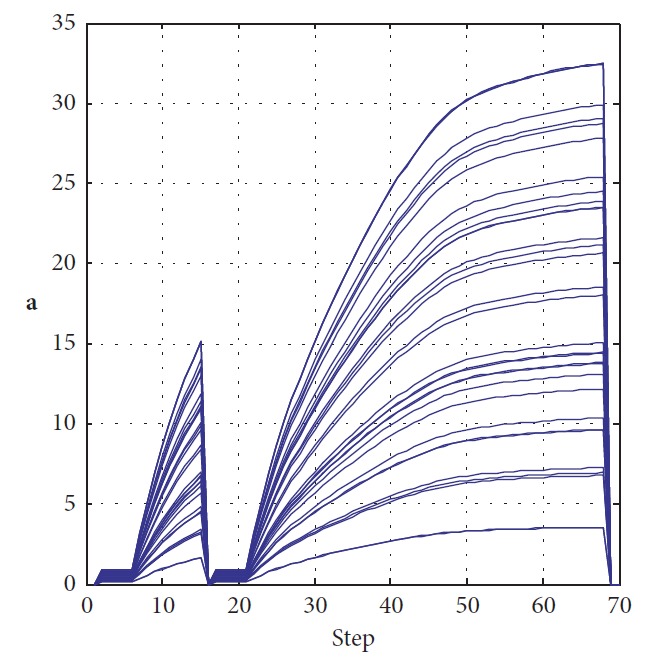
Changes of RFNN weights.

**Figure 22:**
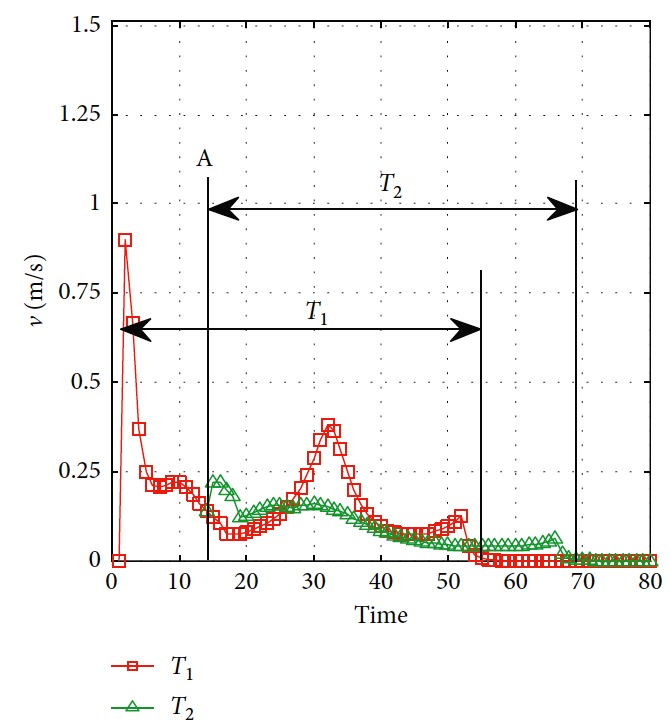
Linear velocity of the mobile robot.

**Figure 23:**
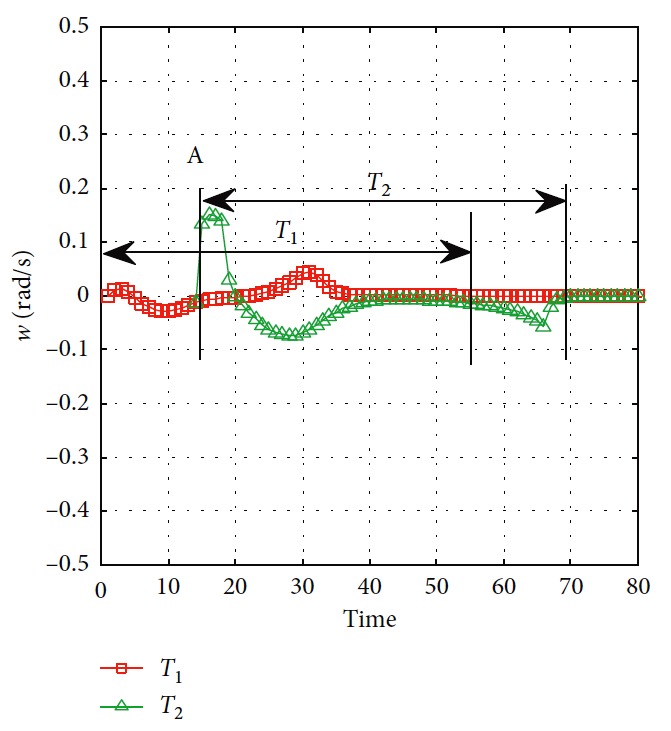
Angular velocity of the mobile robot.

**Figure 24:**
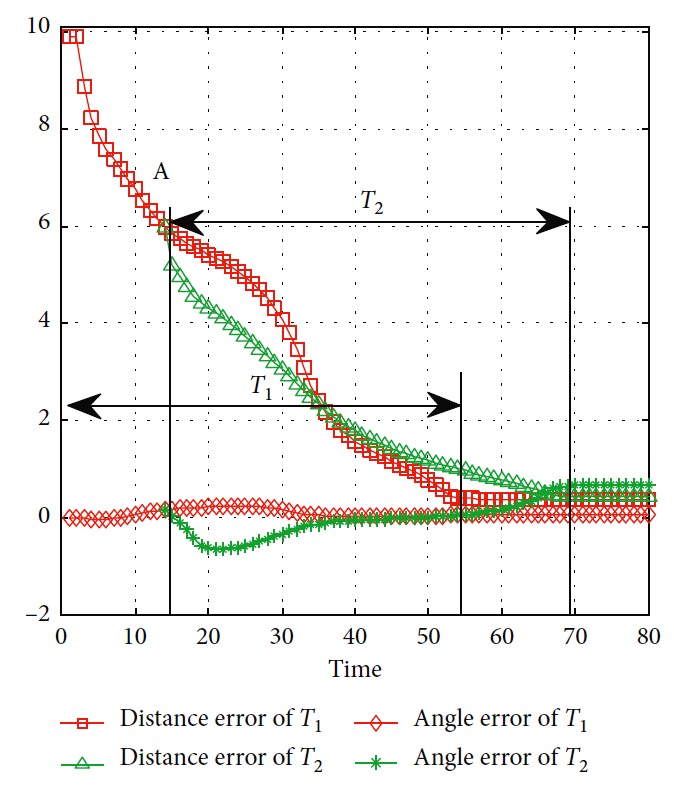
Error of the mobile robot.

Effectiveness of computation, continuity of motion, and smoothness of the predicted trajectory make the proposed planning method feasible and stable to update online. By applying update strategy to RFNN, robust optimality of prediction is guaranteed during the whole process. The performance is shown in Figure 19. The update period is Δ*T* which depends on *σ*. Optimal trajectory during *T*_1_ is generated at the beginning. The position of the target and obstacles keeps changing. If the autonomous mobile robot keeps moving according to trajectory 1, collision will happen. Threshold settled in (37) is reflected in Figure 20. Then, weights of RFNN are retrained as shown in Figure 21. Optimal trajectory during *T*_2_ is generated considering the updated position of obstacles and target. Then robot changes the route at point A in Figure 19. Corresponding changes of linear velocity is shown in Figure 22 at point A. Contrasted to linear velocity, change of angular velocity is drastic in Figure 23. Because robot needs to avoid collision. Position error during whole process is shown in Figure 24. Before A point, red lines represent robot's position error. After point A, the green line represents the robot's position error. More computation periods can be added until the mobile robot reaches the target. This guarantees the real-time optimization.

### 5.6. Hardware Application Based on Omnidirectional Vision

**Figure 25:**
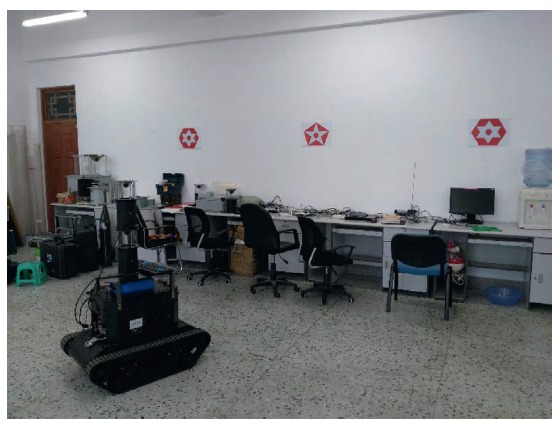
Experiment environment with artificial landmarks.

In order to examine the effectiveness of our planning method, we also implement it to the realistic mobile robot in Figure 25. The visual navigation has attracted many researchers [27]. A catadioptric omnidirectional camera is popularly used in recent years for its advantage of wide field of view [28]. It can capture horizontal field of view in a single image. So, the vision system based on the catadioptric omnidirectional camera is carried on the mobile robot to percept surroundings in this paper. Detailed imaging theory is introduced in our previous work [29, 30].

**Figure 26:**
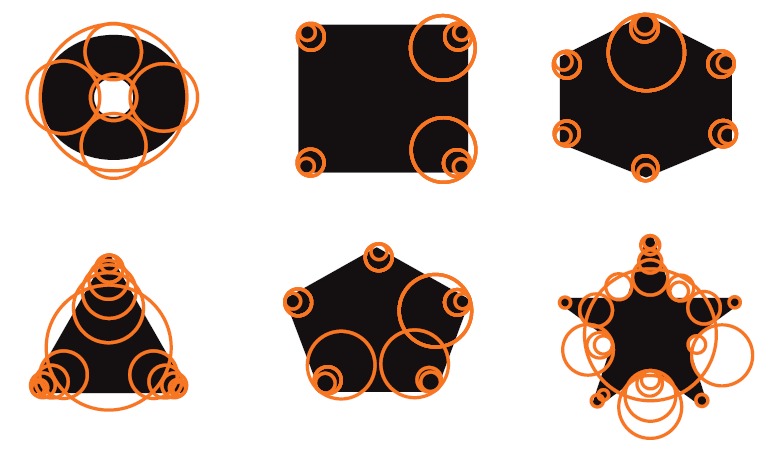
SURF of the sample landmarks.

Although it is a binocular stereo vision system, we only use the lower camera of it because binocular stereo omnidirectional vision takes up much computation resource and runtime performance is not good in real-time application. When the mobile robot moves fast, binocular structure swings violently. In order to use monocular vision to achieve location, artificial landmarks are designed and SURF (speeded-up robust features) are extracted to guarantee rotation and scale invariance. The features of simple shapes are extracted firstly as shown in Figure 26. The landmarks are then designed using these shapes and are placed on the wall forming the location system as shown in Figure 25. Detailed introduction of the location method is introduced in [31, 32].

**Figure 27:**
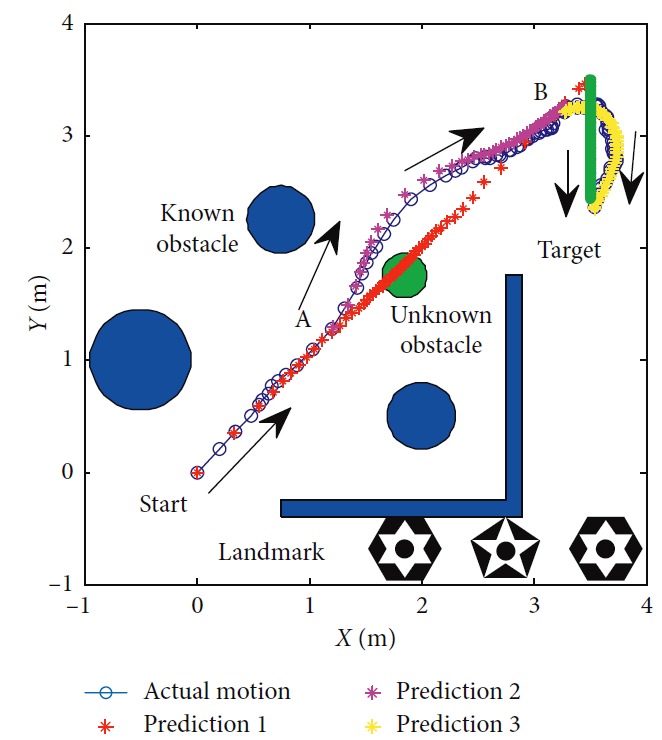
Motion trajectory of the mobile robot.

**Figure 28:**
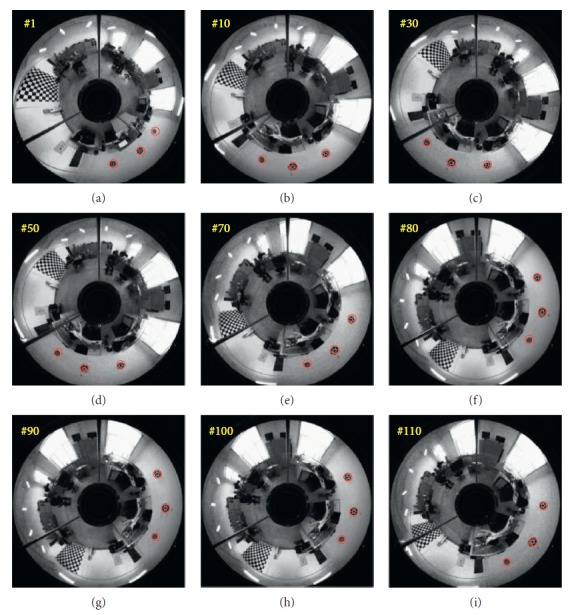
Monocular omnidirectional vision system.

**Figure 29:**
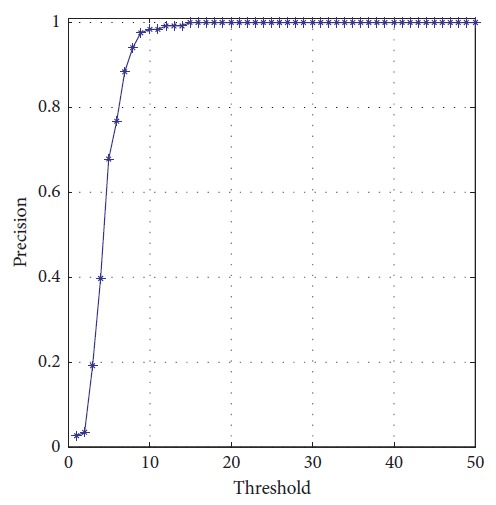
Landmark matching precision during process.

**Figure 30:**
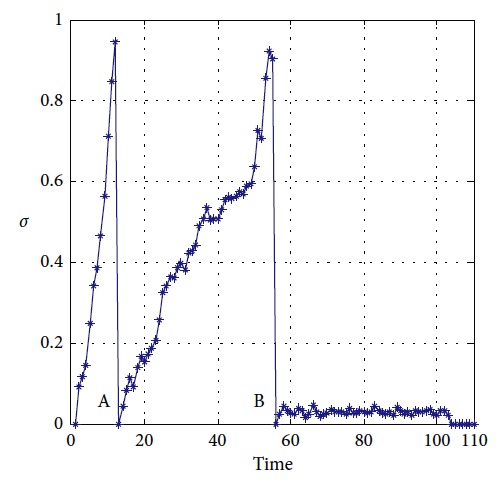
Changes of threshold during process.

**Figure 31:**
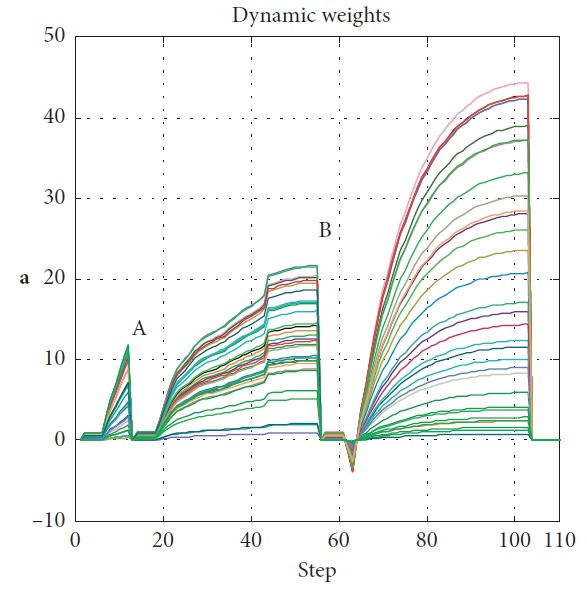
Changes of RFNN weights during process.

**Figure 32:**
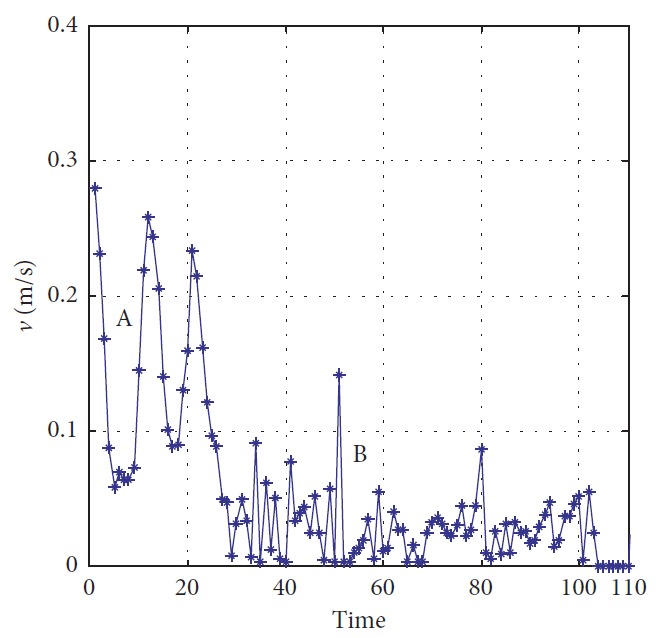
Linear velocity of the mobile robot.

**Figure 33:**
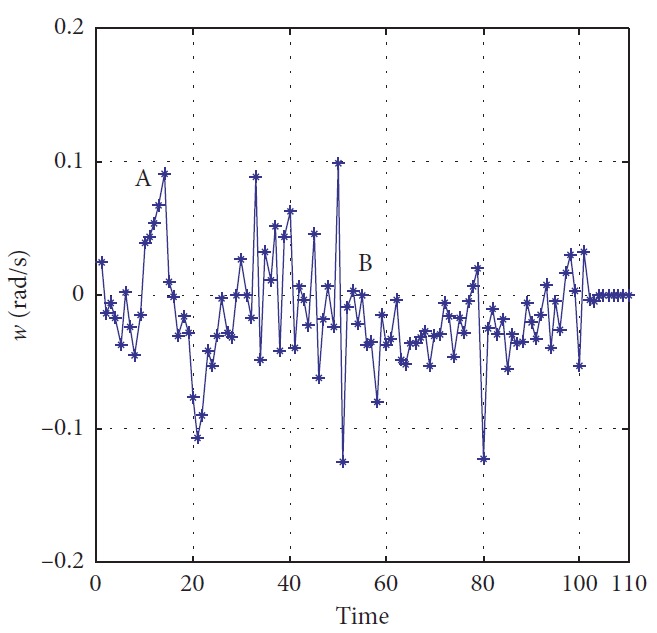
Angular velocity of the mobile robot.

**Figure 34:**
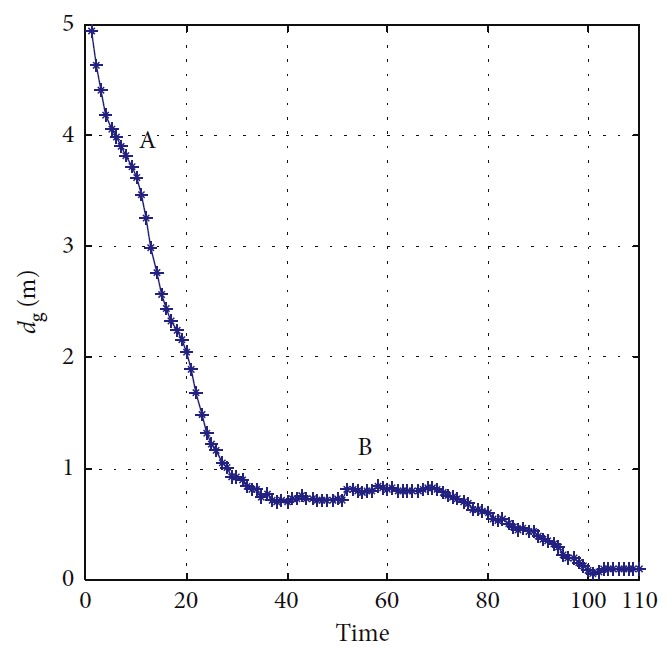
Position error of the mobile robot.

The planning method proposed in this paper is carried out, and the performance of mobile robot is shown in Figure 27. The obstacles in blue are known to the mobile robot, and the green one is unknown. The target position keeps changing during the process. There are totally three predictions. One is generated at the beginning in red. After the unknown obstacle is detected, prediction 2 in magenta is generated at point A. Prediction 3 is generated to lead the mobile robot to reach the final station of the target at the B point. The blue circles represent the actual motion of the mobile robot. Performance of the vision system is shown in Figure 28. Detailed information of the prediction and actual motion is listed in Table 8. During the process, the robot locates itself by tracking the three landmarks. The landmarks matching the precision curve in Figure 29 shows the percentage of correctly tracked frames for a range of distance thresholds. Precision is 98.3% at 10 pixels. Update of trajectory is determined by settled threshold in (36) and (37). If *σ* is beyond threshold, weights of RFNN are retrained as mentioned above. Changes of *σ* are illustrated in Figure 30. *σ* is beyond threshold at A and B. It leads to update of RFNN weights as shown in Figure 31. Robot's motion is then changed in Figures 32 and 33.  Figure 34 reflects the position error of the mobile robot. In actual application, maximum velocities are limited for safety. According to hardware experiment, our method's effectiveness is proved.

Among all experiment results above, the mobile robot's motion is drastic at the beginning. This is reflected by linear velocity. The reason is that weights of RFNN are generated randomly at the beginning. After learning using EKF, predicted motion of the mobile robot is stable. Experiments are carried out in limited area due to limited perception ability of the vision system. But, it can be popularized to large-scale navigation by combining the proposed program method with the topological mapping method introduced in [33].

## 6. Conclusion

By designing a simple RFNN structure and using an effective EKF-based learning method, a novel motion planning strategy is introduced. The greatest novelty is that the proposed method plans both motion and trajectory in real time. Robust optimization of solution is guaranteed. According to simulation and hardware experiments, effectiveness is proved. All the experiments in this paper are limited in a small area. Contrasted to path planning in autonomous driving area, it is a kind of a local planning method. Furthermore, the characteristics of planning in free-space make it a suitable supplement to other global methods. It can also be used as an obstacle avoidance method. Although the method is introduced by considering the unmanned ground mobile robot in 2D condition, it can be popularized to unmanned aircraft and underwater unmanned vehicle in 3D condition. Because our method does not need the previous map and offline computation, it takes up less memory space in contrast to other methods. Thus, it may have good performance in 3D condition where surroundings are complex. In order to improve runtime performance, initial weights of RFNN are generated randomly, which can be modified in future work. Some heuristic methods can be designed to choose initial weights of RFNN. The structure of RFNN used in this paper is simple. The output of RFNN is not considered as the input of RFNN. More complex recurrent structures can be designed to improve effectiveness of the RFNN planning strategy in the future. Furthermore, the location method of the autonomous mobile robot used in this paper is too simple. It also needs to be improved in future. Simultaneous localization and mapping (SLAM) and visual-inertial odometry techniques will be designed in our system. The perception system will be developed more effectively in the future.

## Figures and Tables

**Table 1:** Initialization of the parameters.

*b*	0.2
**c**	$\left[\begin{array}{cccc} 0 & 0 & -\left(\pi /2\right) & -\left(\pi /2\right)\\ 10 & 5 & \pi /2 & \pi /2 \end{array}\right]$
**σ**	$\left[\begin{array}{cccc} 2.5 & 1 & 1 & 1 \end{array}\right]$
*λ*	0.3
*α*	0.3
**q**_max_	${\left[\begin{array}{cc} 1 & 1 \end{array}\right]}^{T}$
Δ_*t*_	0.5
Δ_*o*_	0.3
*σ*_bound_	0.5
Δ*t*	1

**Table 2:** Trajectory information.

	Distance (m)	Step	Cost (s)	Error (m)
Trajectory 1	10.20	62	0.228	0.389
Trajectory 2	10.44	59	0.347	0.303

**Table 3:** RFNN weights of trajectory 1.

*k*=1	*k*=10	*k*=20	*k*=30	*k*=40	*k*=45	*k*=50
0.701	5.396	15.40	20.60	23.32	24.49	24.82
0.209	1.616	4.614	6.174	6.986	7.337	7.437
0.533	4.110	11.73	15.69	17.76	18.65	18.90
0.359	2.772	7.913	10.58	11.98	12.58	12.75
0.713	5.493	15.67	20.97	23.73	24.93	25.26
0.007	0.053	0.152	0.204	0.230	0.242	0.245
0.639	4.926	14.06	18.81	21.28	22.35	22.66
0.126	0.972	2.775	3.713	4.202	4.413	4.473
0.774	5.965	17.02	22.78	25.78	27.07	27.44
0.537	4.138	11.81	15.80	17.88	18.78	19.03
⋮	⋮	⋮	⋮	⋮	⋮	⋮
0.220	1.910	5.496	8.076	9.053	9.406	9.448

**Table 4:** RFNN weights of trajectory 2.

*k*=1	*k*=10	*k*=20	*k*=30	*k*=40	*k*=45	*k*=50
0.101	0.882	2.538	3.730	4.181	4.344	4.363
0.756	6.545	18.82	27.66	31.01	32.22	32.36
0.866	7.494	21.55	31.67	35.50	36.89	37.05
0.996	8.624	24.80	36.45	40.86	42.45	42.64
0.098	0.852	2.451	3.601	4.037	4.195	4.213
0.172	1.493	4.295	6.312	7.075	7.351	7.383
0.484	4.192	12.05	17.72	19.86	20.64	20.73
0.937	8.110	23.32	34.28	38.42	39.92	40.10
0.509	4.409	12.68	18.63	20.89	21.70	21.80
⋮	⋮	⋮	⋮	⋮	⋮	⋮
0.466	4.036	11.61	17.06	19.12	19.87	19.95

**Table 5:** Information of trajectories.

	Distance (m)	Step	Cost	Terminal error
1	9.936	76	0.091	0.439
2	14.72	87	0.059	0.483
3	9.866	96	0.022	0.402
4	10.01	74	0.038	0.221
5	12.13	62	0.129	0.471
6	9.744	75	0.102	0.384
7	10.15	78	0.067	0.331
8	9.683	58	1.342	0.369
9	10.36	50	0.089	0.463
10	9.957	65	0.011	0.451

**Table 6:** Trajectory information.

	Distance (m)	Step	Cost (s)	Error (m)
Opti	9.677	60	37.891	0.328
A^*∗*^	9.838	62	0.512	0.354
RFNN	9.683	58	1.342	0.369

**Table 7:** Trajectory information.

	Distance (m)	Step	Error (m)	State
State 1	9.683	58	0.369	Success
State 2	9.699	50	0.374	Success
State 3	4.714	15	4.723	Fail

**Table 8:** Trajectory information.

	Distance (m)	Step	Error (m)	Cost (s)
Prediction 1	4.747	57	0.155	1.261
Prediction 2	3.574	47	0.174	0.514
Prediction 3	1.274	52	0.123	0.081
Actual motion	6.569	103	0.099	110

## Data Availability

The data used to support the findings of this study are available from the corresponding author upon request.

## References

[1] Rosolia U., De Bruyne S., Alleyne A. G. (2017). Autonomous vehicle control: a nonconvex approach for obstacle avoidance. *IEEE Transactions on Control Systems Technology*.

[2] Faigl J. (2016). An application of self-organizing map for multirobot multigoal path planning with minmax objective. *Computational Intelligence and Neuroscience*.

[3] Ni J., Wu L., Shi P., Yang S. X. (2017). A dynamic bioinspired neural network based real-time path planning method for autonomous underwater vehicles. *Computational Intelligence and Neuroscience*.

[4] Li Q., Chen L., Li M., Shaw S.-L., Nuchter A. (2014). A sensor-fusion drivable-region and lane-detection system for autonomous vehicle navigation in challenging road scenarios. *IEEE Transactions on Vehicular Technology*.

[5] Majumdar A., Tedrake R. (2017). Funnel libraries for real-time robust feedback motion planning. *International Journal of Robotics Research*.

[6] Mac T. T., Copot C., Tran D. T., De Keyser R. (2016). Heuristic approaches in robot path planning: a survey. *Robotics and Autonomous Systems*.

[7] Montiel O., Sepúlveda R., Orozco-Rosas U. (2014). Optimal path planning generation for mobile robots using parallel evolutionary artificial potential field. *Journal of Intelligent & Robotic Systems*.

[8] Montiel O., Orozco-Rosas U., Sepúlveda R. (2015). Path planning for mobile robots using Bacterial Potential Field for avoiding static and dynamic obstacles. *Expert Systems with Applications*.

[9] Gonzalez D., Perez J., Milanes V., Nashashibi F. (2016). A review of motion planning techniques for automated vehicles. *IEEE Transactions on Intelligent Transportation Systems*.

[10] Vorobieva H., Glaser S., Minoiu-Enache N., Mammar S. Automatic parallel parking with geometric continuous-curvature path planning.

[11] Karami A. H., Hasanzadeh M. (2015). An adaptive genetic algorithm for robot motion planning in 2D complex environments. *Computers & Electrical Engineering*.

[12] Abdessemed F., Faisal M., Emmadeddine M. (2014). A hierarchical fuzzy control design for indoor mobile robot. *International Journal of Advanced Robotic Systems*.

[13] Wang M., Liu J. N. K. (2008). Fuzzy logic-based real-time robot navigation in unknown environment with dead ends. *Robotics and Autonomous Systems*.

[14] Singh M. K., Parhi D. R. (2011). Path optimisation of a mobile robot using an artificial neural network controller. *International Journal of Systems Science*.

[15] Dierks T., Brenner B., Jagannathan S. (2013). Neural network-based optimal control of mobile robot formations with reduced information exchange. *IEEE Transactions on Control Systems Technology*.

[16] Peng Z., Wen G., Yang S., Rahmani A. (2016). Distributed consensus-based formation control for nonholonomic wheeled mobile robots using adaptive neural network. *Nonlinear Dynamics*.

[17] Juang C. F., Huang R. B., Lin Y. Y. (2009). A recurrent self-evolving interval type-2 fuzzy neural network for dynamic system processing. *IEEE Transactions on Fuzzy Systems*.

[18] Lin Y. Y., Chang J. Y., Lin C. T. (2013). Identification and prediction of dynamic systems using an interactively recurrent self-evolving fuzzy neural network. *IEEE Transactions on Neural Networks and Learning Systems*.

[19] Kim C.-J., Chwa D. (2015). Obstacle avoidance method for wheeled mobile robots using interval type-2 fuzzy neural network. *IEEE Transactions on Fuzzy Systems*.

[20] Jolly K. G., Sreerama Kumar R., Vijayakumar R. (2010). Intelligent task planning and action selection of a mobile robot in a multi-agent system through a fuzzy neural network approach. *Engineering Applications of Artificial Intelligence*.

[21] Khanesar M. A., Kayacan E., Teshnehlab M., Kaynak O. (2012). Extended Kalman filter based learning algorithm for type-2 fuzzy logic systems and its experimental evaluation. *IEEE Transactions on Industrial Electronics*.

[22] Rubio J. J., Yu W. (2007). Nonlinear system identification with recurrent neural networks and dead-zone Kalman filter algorithm. *Neurocomputing*.

[23] Wang X., Huang Y. (2011). Convergence study in extended kalman filter-based training of recurrent neural networks. *IEEE Transactions on Neural Networks*.

[24] Mitsch S., Ghorbal K., Vogelbacher D., Platzer A. (2017). Formal verification of obstacle avoidance and navigation of ground robots. *International Journal of Robotics Research*.

[25] Han Y., Zhu Q., Xiao Y. Data-driven control of autonomous vehicle using recurrent fuzzy neural network combined with PID method.

[26] Puskorius G. V., Feldkamp L. A. (1994). Neurocontrol of nonlinear dynamical systems with Kalman filter trained recurrent networks. *IEEE Transactions on Neural Networks*.

[27] Font F. B., Ortiz A., Oliver G. (2008). Visual navigation for mobile robots: a survey. *Journal of Intelligent & Robotic Systems*.

[28] Cinaroglu I., Bastanlar Y. (2015). A direct approach for object detection with catadioptric omnidirectional cameras. *Signal, Image and Video Processing*.

[29] Zhu Q., Liu C., Cai C. (2015). A novel robot visual homing method based on SIFT features. *Sensors*.

[30] Cai C., Fan B., Weng X., Zhu Q., Su L. (2017). A target tracking and location robot system based on omnistereo vision. *Industrial Robot: An International Journal*.

[31] Zhu Q., Xie H., Cai C., Liu P. A rapid and precise self-localization approach of mobile robot based on binocular omni-directional vision.

[32] Zhu Q., Liu P., Cai C. (2017). Robust method of indoor robot localization based on artificial landmark. *Journal of Computer Applications*.

[33] Zhu Q., Liu X., Cai C. (2014). Feature optimization for long-range visual homing in changing environments. *Sensors*.

